# 4,4-Dimethylsterols Reduces Fat Accumulation via Inhibiting Fatty Acid Amide Hydrolase In Vitro and In Vivo

**DOI:** 10.34133/research.0377

**Published:** 2024-05-29

**Authors:** Tao Zhang, Liangliang Xie, Yiwen Guo, Zhangtie Wang, Xin Guo, Ruijie Liu, Qingzhe Jin, Ming Chang, Xingguo Wang

**Affiliations:** ^1^ School of Food Science and Technology, State Key Laboratory of Food Science and Technology, Jiangnan University, Wuxi 214122, China.; ^2^ College of Food Science and Technology, Huazhong Agricultural University, Wuhan 430070, China.; ^3^ School of Biological and Food Engineering, Anhui Polytechnic University, Wuhu 241000, China.; ^4^ College of Biosystems Engineering and Food Science, Zhejiang University, Hangzhou 310058, China.; ^5^ Department of Food Science, University of Massachusetts Amherst, Amherst, MA 01003, USA.

## Abstract

4,4-Dimethylsterols constitute a unique class of phytosterols responsible for regulating endogenous cannabinoid system (ECS) functions. However, precise mechanism through which 4,4-dimethylsterols affect fat metabolism and the linkage to the ECS remain unresolved. In this study, we identified that 4,4-dimethylsterols, distinct from 4-demethseterols, act as inhibitors of fatty acid amide hydrolases (FAAHs) both in vivo and in vitro. Genetic ablation of FAAHs (*faah-1*) abolishes the effects of 4,4-dimethylsterols on fat accumulation and locomotion behavior in a *Caenorhabditis elegans* model. We confirmed that dietary intervention with 4,4-dimethylsterols in a high-fat diet (HFD) mouse model leads to a significant reduction in body weight (>11.28%) with improved lipid profiles in the liver and adipose tissues and increased fecal triacylglycerol excretion. Untargeted and targeted metabolomics further verified that 4,4-dimethylsterols influence unsaturated fatty acid biosynthesis and elevate oleoyl ethanolamine levels in the intestine. We propose a potential molecular mechanism in which 4,4-dimethylsterols engage in binding interactions with the catalytic pocket (Ser241) of FAAH-1 protein due to the shielded polarity, arising from the presence of 2 additional methyl groups (CH_3_). Consequently, 4,4-dimethylsterols represent an unexplored class of beneficial phytosterols that coordinate with FAAH-1 activity to reduce fat accumulation, which offers new insight into intervention strategies for treating diet-induced obesity.

## Introduction

Cholesterol biosynthesis from lanosterol in mammalian cells is a lengthy and energy-consuming pathway. Despite the existence of a diverse range of sterols, cholesterol was the major steroid molecular chosen as a major component of mammalian cell membranes during the evolution of life [1]. This choice can be attributed to the presence of methyl groups (CH_3_) at the carbon-4 atom in the sterol A-ring, such as lanosterol, which strikingly alters its interaction with the membrane phospholipid bilayer [2]. Therefore, sterols with 2 CH_3_ substituents at the carbon-4 position are classified as 4,4-dimethylsterols (DMS) [3]. DMS are commonly consumed in our daily diet through vegetable oils, nuts, and cereals. Furthermore, phytosterols have been recognized as safe by several international authorities, including the European Commission, the Food and Drug Administration (FDA), and the Ministry of Health of China [4]. In our previous studies, we conducted multiple investigations to understand various aspects of DMS, including structural identifications [5], chemical properties [6], and potential health benefits [7–9]. We reported a possible correlation between DMS intake and noncommunicable diseases (NCDs), suggesting that higher DMS intake is associated with lower mortality from NCDs [10]. In-depth investigations have ascertained that DMS is an effective inhibitor of stearoyl-CoA desaturase (SCD), which contributes to improved lipid metabolism [8,11]. Given the pivotal role of the endogenous cannabinoid system (ECS) in the occurrence of NCDs [12], particularly its impact on lipid metabolism through interactions with the SCD [13], we propose that the ECS is involved in the DMS-mediated lipid metabolism machinery, which remains unresolved.

The ECS is a crucial lipid signaling system comprising 2 G protein-coupled receptors, lipid ligands, and enzymes responsible for ligand synthesis [14]. Fatty acid amide hydrolase (FAAH) is a key enzyme within the ECS and plays an important role in appetite regulation and lipid metabolism. Reduced FAAH activity has been demonstrated to lower food intake [15], increase energy expenditure [16], and improve insulin sensitivity [17], all of which contribute to the development of abnormal fat accumulation and obesity. Obesity has become one of the most severe global NCDs and has reached pandemic levels. Both animal and human studies have suggested that targeting FAAH activity is a potential therapeutic strategy for treating obesity [18,19]. In addition to the known interactions between DMS and the ECS [10], it is of interest to further elucidate how DMS interferes with FAAH activity and the consequences of their interplay in lipid metabolism.

*Caenorhabditis elegans* is a model organism that shares similar fat regulatory pathways and homologs of human genes involved in lipid metabolism [20]. *C. elegans* is highly esteemed as an animal model in obesity research due to its well-characterized genetic tools. Over the past decade, studies utilizing *C. elegans* have significantly advanced our understanding of body lipid metabolism and have contributed substantially contributed to our knowledge of various diseases, particularly in the nutritional evaluation of bioactive constituents in food [21].

Here, we aimed to investigate the effects of DMS on FAAH activity using various models, including *C. elegans*, THLE-2 cells, and a mouse model of high-fat diet (HFD)-induced obesity. We conducted a comparative analysis with 4-demethylsterols due to their distinct structural features, which lack methyl groups at the carbon-4 position but are widely recognized for their cholesterol-lowering properties [22]. Our findings offer initial insights into the distinct mechanisms by which DMS reduces fat accumulation compared to 4-demethylsterols. Importantly, we have confirmed that DMS acts as a specific FAAH inhibitor, both in vivo and in vitro. This study underscores the potential of DMS as an unexplored functional phytosterol with promise for preventing obesity development in the future.

## Results

### DMS reduces fat accumulation and enhances locomotion in *C. elegans*

**Fig. 1.**
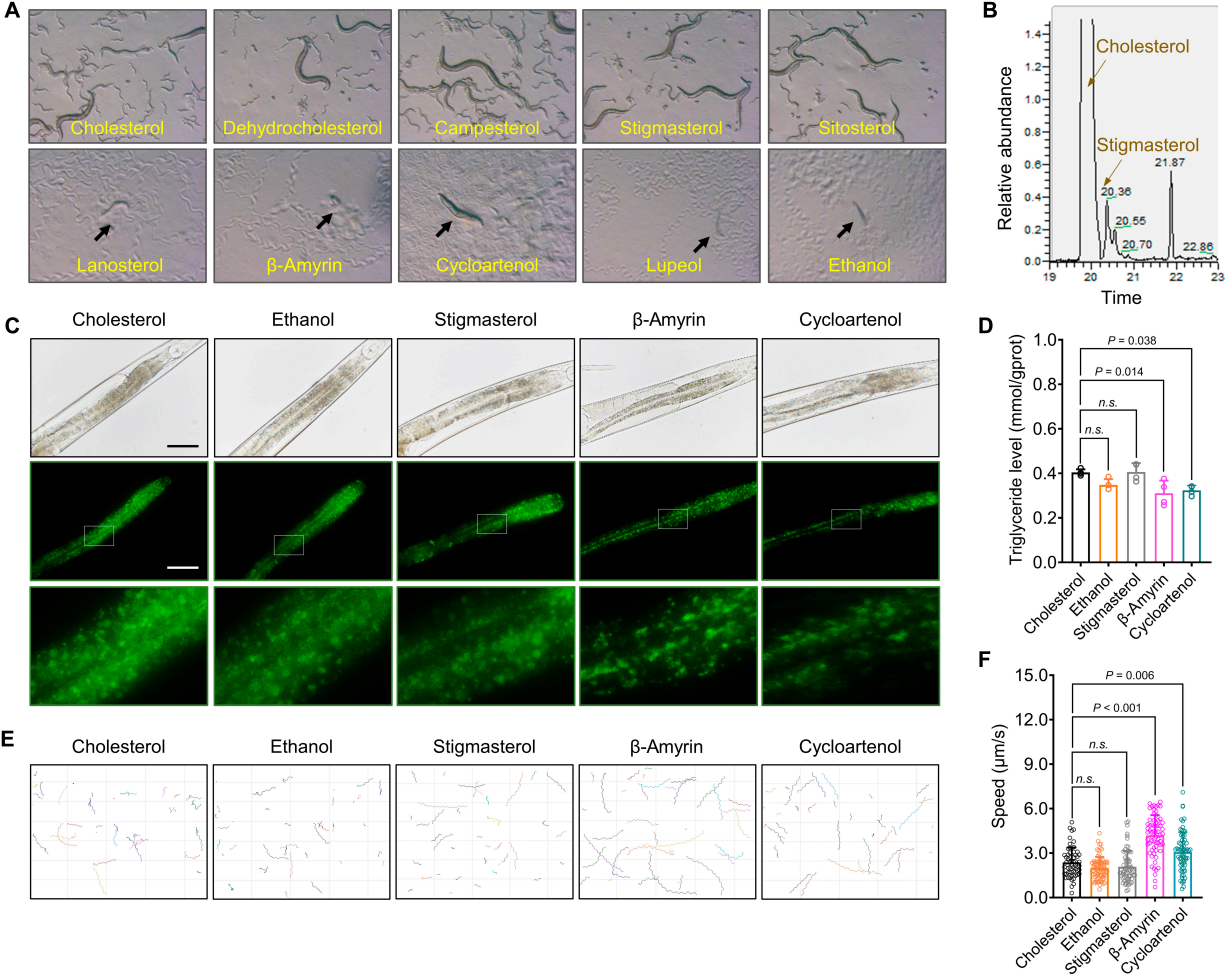
DMS suppresses cholesterol-depleted *C. elegans* growth but reduces fat accumulation and enhances locomotion in the presence of cholesterol. (A) DMS (lanosterol, β-amyrin, cycloartenol, and lupeol) fails to support the population growth in the third cholesterol-depleted generation of worms. (B) Cholesterol synthesis occurs in wild-type *C. elegans* when fed with 4-demethylsterol (stigmasterol) as analyzed by GC-MS analysis. (C) Dietary feeding of DMS in the presence of cholesterol reduces fat accumulation as indicated by lower GFP fluorescence intensity in worms carrying GFP-tagged lipid droplets (scale bar: 50 μm). (D) DMS treatment decreases triglycerides (TG) levels measured by using a TG assay kit. (E and F) Dietary feeding of DMS in the presence of cholesterol increases moving distance (E) and enhances moving speed (F) as analyzed by WormLab tracker software. Significant differences among 3 or more mean values were determined using one-way ANOVA with Tukey’s multiple comparisons test.

In the nematode *C. elegans*, many phytosterols serve as precursors to the steroid hormone dafachronic acid, which plays an important role in *C. elegans* development [23]. Prior to investigating the function of DMS in the *C. elegans* model, we examined whether DMS could substitute for cholesterol and support population growth in the third generation of cholesterol-depleted worms. Compared to cholesterol and 4-desmethylsterols (campesterol, stigmasterol, and sitosterol), supplementation with DMS (lanosterol, β-amyrin, cycloartenol, and lupeol) failed to support population growth (Fig. 1A). In *daf-36* mutant worms, which exhibit defects in the cholesterol synthesis pathway, supplementation solely with 7-dehydrocholesterol rescued development (Fig. S1A). This observation is consistent with cholesterol being synthesized in both wild-type worms fed with stigmasterol and daf-36 mutant worms fed with 7-dehydrocholesterol (Fig. 1B and Fig. S1B). The results indicate that DMS cannot serve as an alternative substitute for cholesterol in *C. elegans*, unlike 4-demethylsterols. DMS cannot be converted into cholesterol, leading to arrested development (Fig. S1C). A previous study also reported that *C. elegans* lacks the capacity to remove methyl groups (CH_3_) from the carbon-4 position [24]. The results suggest that DMS supplementation alone does not yield any intermediate compounds in the downstream synthesis pathway of 7-dehydrocholesterol, indicating that DMS is not involved in sterol auxotrophy in *C. elegans*.

Subsequently, DMS was supplemented with cholesterol to examine its effects on fat accumulation and locomotion. When treated with DMS, *C. elegans* expressing green fluorescent protein (GFP)-tagged lipid droplets exhibited a significant reduction in fluorescence intensity, indicating decreased fat accumulation (Fig. 1C). Using a triglyceride assay kit as an alternative measurement, treatment with cycloartenol and β-amyrin remarkably reduced triglyceride levels by 19.73% and 23.03%, respectively (Fig. 1D). No significant difference was observed between stigmasterol treatment and the ethanol control. Moving behavior analysis revealed that DMS significantly increased the moving distance and average speed compared to controls fed with 4-demethylsterols (stigmasterol) (Fig. 1E and F). Thus, DMS reduces fat accumulation and enhances motility without interfering with the cholesterol auxotrophic pathway in *C. elegans*.

### DMS inhibits FAAH-1 activity in vivo and in vitro

**Fig. 2.**
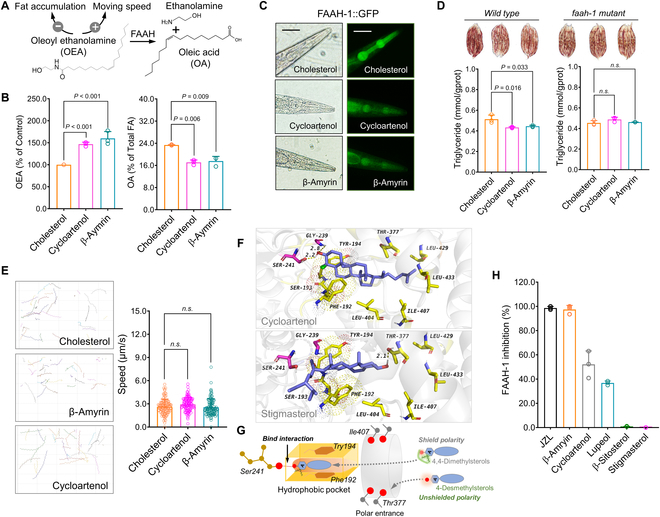
DMS exhibits inhibitory effect on FAAH-1 activity in *C. elegans* and in vitro. (A) OEA is a signal lipid involved in fat accumulation and locomotion. (B) Supplementation of DMS (cycloartenol and β-amyrin) leads to an increase in OEA levels and a reduction in OA levels in *C. elegans*. (C) Cycloartenol and β-amyrin decrease FAAH-1 expression in the pharynx by using FAAH-1::GFP transgenic worms (scale bar: 50 μm). (D and E) Genetic ablation of *faah-1* abolished the effects of DMS on fat accumulation (D) and average moving speed (E). (F) An example of docking result between cycloartenol and FAAH-1 protein after molecular docking screening of various phytosterols using AutoGrid4 and AutoDock4 software. (G) Proposed mechanism of FAAH-1 inhibition by DMS via bonding to Ser241 residue. (H) Inhibitory activity assay by using purified human recombinant FAAH-1 enzyme confirms DMS as inhibitors of FAAH-1 in vitro. Significant differences among 3 or more mean values were determined using one-way ANOVA with Tukey’s multiple comparisons test.

In *C. elegans*, fatty acid amide hydrolase-1 (FAAH-1) is involved in reducing fat accumulation and increasing locomotion [25]. We hypothesized that the decrease in fat levels and increase in motility may result from an interaction between DMS and FAAH-1. As shown in Fig. 2A, FAAH-1 catalyzes the hydrolysis of oleoyl ethanolamine (OEA), enhancing the hydrolysis to oleic acid (OA). We developed a targeted multiple-reaction-monitoring method to measure OEA levels in *C. elegans* (Fig. S2A). The OEA level, but not the OA level, was significantly increased by DMS (cycloartenol and β-amyrin) (Fig. 2B). Subsequently, we employed transgenic *C. elegans* expressing FAAH-1::GFP to measure FAAH-1 expression. Compared to the cholesterol control, a lower fluorescence intensity in the pharynx was observed in both cycloartenol- and β-amyrin-treated worms, confirming a reduction in FAAH-1 expression by DMS (Fig. 2C). Using a *faah-1* deletion mutant (TM5011) [26], we found that the reduced fat accumulation caused by DMS in wild-type worms was abolished in TM5011 worms (Fig. 2D). Furthermore, the moving distance and speed also showed no significant differences in the *faah-1* deletion mutant (Fig. 2E). These results suggest that DMS interferes with FAAH-1 activity, affecting both fat accumulation and locomotion. Indeed, the *faah-1* mRNA level was also reduced by DMS treatment (Fig. S2B).

To explore how different phytosterols affect FAAH-1 activity, we utilized molecular docking to examine differences among various phytosterols. Interestingly, unlike 4-demethylsterols, DMS was capable of inserting into the active catalytic site (Ser241), a residue crucial for FAAH-1 activity (Fig. 2F and G and Fig. S2C). A hydrogen bond interaction (2.2 Å) was observed between Ser241 and cycloartenol. In contrast, 4-desmethylsterols only exhibited binding interactions with the THR-377 residue, located at the surface entrance of FAAH-1. For instance, stigmasterol and sitosterol formed unfavorable hydrogen bonds (2.1 Å and 1.9 Å) with Thr377 (Fig. 2F and G). The docking results confirmed that the inhibitory activity against FAAH-1 is likely due to the binding interaction between DMS and the active pocket (Ser241) within the FAAH-1 protein. Indeed, an inhibitory assay using purified FAAH-1 protein confirmed the docking results (Fig. 2H). The activity of FAAH-1 was suppressed not only by cycloartenol and β-amyrin but also by other DMS such as lupeol. However, 4-demethylsterols (stigmasterol and β-sitosterol) did not exhibit significant inhibitory effects on FAAH-1 activity (Fig. 2H). Therefore, DMS affects both FAAH-1 expression and FAAH-1 activity.

### Purified dietary DMS reduces triacylglycerols in cell model

**Fig. 3.**
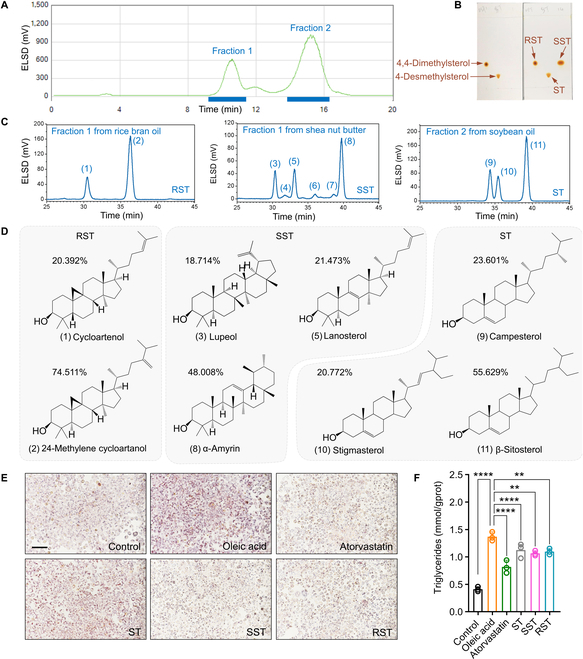
Purified DMS from dietary oils reduces fat accumulation in cell models. Isolation (A), purity validation (B), and composition analysis (C) of different phytosterols by using MPLC, TLC, and HPLC-ELSD. (D) Structures and percentages of major phytosterols in RST, SST, and ST. (E) Oil red O staining of THLE-2 cells treated with different classes of phytosterols (ST, SST, and RST). (F) DMS supplementation reduces triglycerides accumulation in THLE-2 cells. Significant differences among 3 or more mean values were determined using one-way ANOVA with Tukey’s multiple comparisons test.

Considering that DMS exhibits an inhibitory effect on FAAH-1 activity and reduces fat accumulation in *C. elegans*, we sought to investigate whether DMS from our dietary source could confer similar beneficial effects in other models. To address this question, we employed medium-pressure liquid chromatography to purify the dietary DMS present in edible vegetable oils, a major dietary source of DMS according to our previous study [5]. As shown in Fig. 3A, fraction 1 and fraction 2 represent DMS and 4-desmethylsterols, respectively. Fraction 1 is collected from the unsaponifiable matter of rice bran oil or shea nut butter, while fraction 2 is derived from the unsaponifiable matter of soybean oil. Each fraction consists of a single type of sterol, as confirmed by thin-layer chromatography (Fig. 3B). Detailed sterol composition in rice bran oil (RST), shea nut butter (SST), and soybean oil (ST) was analyzed using HPLC-ELSD (Fig. 3C and D). RST is composed of DMS with 20.392% cycloartenol (1) and 74.511% 24-methylenecycloartanol (2). SST also consists of DMS with 18.714% lupeol (3), 21.473% lanosterol (5), and 48.008% α-amyrin (8). ST only contains 4-desmethylsterols, comprising 23.601% campesterol (9), 20.772% stigmasterol (10), and 55.629% β-sitosterol (11). Given the unique phytosterol profiles in different fractions, we proceeded to compare their lipid-lowering effects using the THLE-2 cell line. As shown in Fig. 3E, intracellular fat accumulation was reduced in all sterol treatment groups. Treatment with 0.4 mM ST, RST, and SST resulted in a reduction of triglyceride accumulation by 17.76%, 19.88%, and 22.15%, respectively (Fig. 3F). These results indicate that purified dietary DMS also reduces triacylglycerols in cell models.

### DMS decreases fat accumulation in a mouse model

**Fig. 4.**
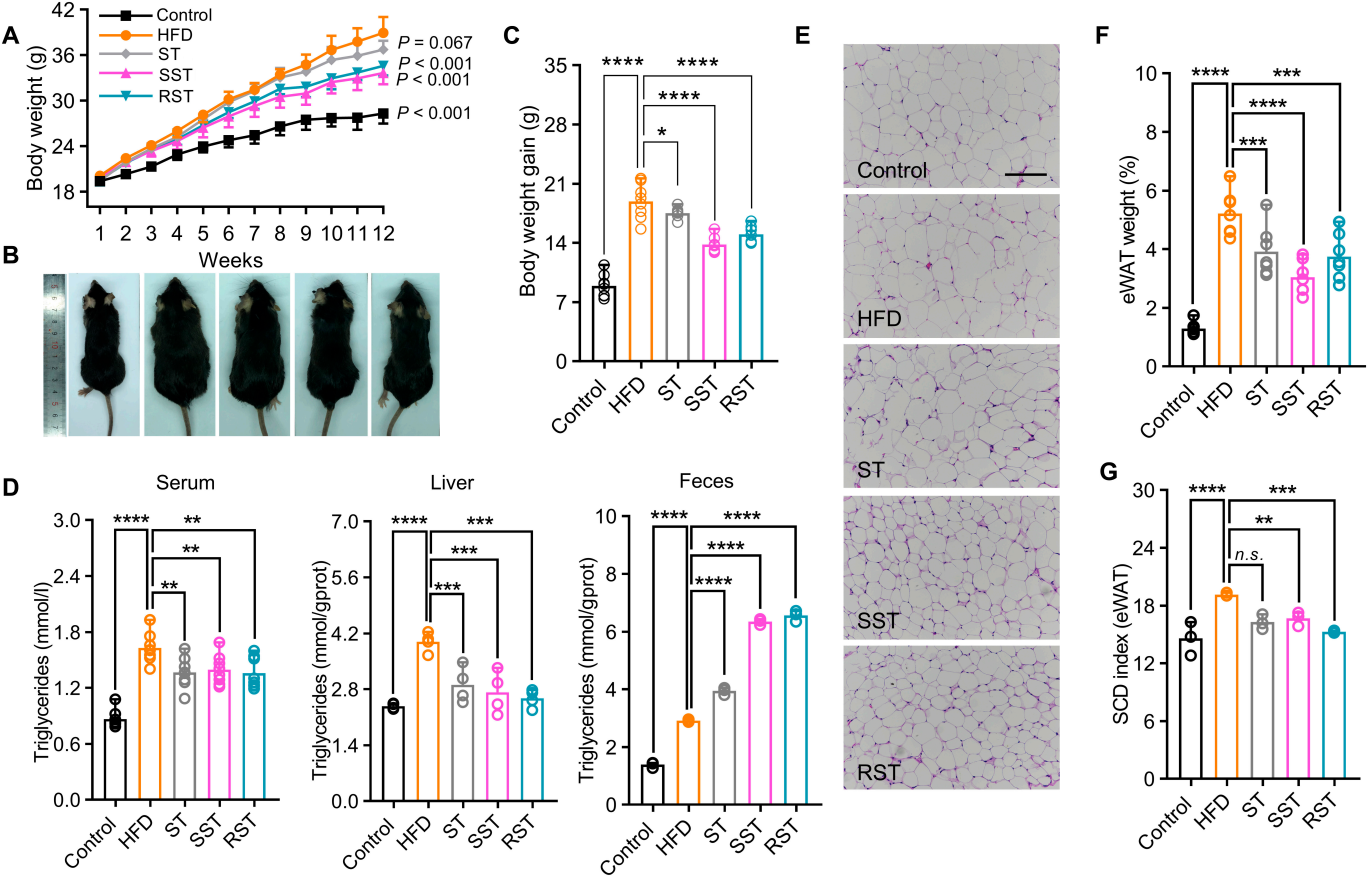
Dietary-derived DMS reduces obesity in a high-fat diet (HFD)-fed mouse model. (A) Body weight of mice fed with normal chow diet (Control), high-fat diet (HFD), and different phytosterols. (B) Representative appearances of mice from each group. (C) DMS significantly reduces body weight gain. (D) DMS decreases triglycerides level in serum and liver while enhancing triacylglycerol excretion in feces. Representative H&E staining (E), weight changes (F), and SCD index (G) of eWAT from different treatment groups (scale bar: 100 μm). Significant differences among 3 or more mean values were determined using one-way ANOVA with Tukey’s multiple comparisons test.

In addition to the cell model, we evaluated the anti-obesity activity of dietary DMS and compared it with 4-demethylsterols using an HFD mouse model. After a 12-week intervention, the body weight of the HFD group was 37.68% higher than that of the control group fed a normal chow diet (Fig. 4A). Representative images of mice from each group at the end of the 12-week intervention are presented in Fig. 4B. Compared to the HFD group, dietary intervention with DMS significantly reduced body weight by 11.28% and 13.72%, respectively (Fig. 4C). Although food intake in the control group was higher than in the HFD group, no significant difference was observed in total energy intake (Fig. S3A and B). Additionally, compared to 4-demethylsterols (ST), DMS supplementation effectively prevented obesity development.

The significant reduction in body weight prompted us to further measure changes in lipid profiles in different tissues. Hepatic histological analysis revealed extensive white vacuolation and severe microvesicular steatosis in the liver of the HFD group compared to the phytosterol intervention groups (Fig. S3C). All intervention groups showed significantly lower liver and serum total triacylglycerol levels (Fig. 4D). We measured fecal triacylglycerol, cholesterol, and phytosterol levels to explore whether fecal lipid excretion contributed to the decreased body weight. Notably, both RST and SST interventions remarkably elevated triacylglycerol excretion compared with the HFD and ST groups (Fig. 4D). In contrast, fecal cholesterol excretion was significantly enhanced in the ST group (Fig. S3D). Only the ST intervention was effective in decreasing total cholesterol levels in the liver (Fig. S3E). Blood glucose levels were also improved by phytosterols (Fig. S3F and G). As triacylglycerol is mainly stored in adipose tissue, we examined whether adipose tissues were affected. Histological analysis of epididymal white adipose tissue (eWAT) showed larger adipocyte size in the HFD group (Fig. 4E). Unlike brown adipose tissue (BAT), eWAT weight decreased significantly when treated with ST, RST, and SST (Fig. 4F and Fig. S3H). Regarding fatty acid profiles, both the proportions of C18:1/C18:0 and C16:1/C16:0 were significantly down-regulated in eWAT when fed with phytosterols, while the proportions in BAT remained stable (Fig. 4G and Fig. S3I and J). These results indicate down-regulated stearoyl-CoA desaturase (SCD) activity in the adipose tissue due to DMS intervention. SCD is considered a potential therapeutic target in obesity-related disorders, and its inhibition contributes to the resistance of diet-induced obesity [27]. Overall, DMS effectively reduces fat accumulation, alleviates lipid profiles in the liver and adipose tissue, and prevents the occurrence of obesity development in mice.

### DMS improves lipid metabolism via enhancing FAAH substrate level in intestine

**Fig. 5.**
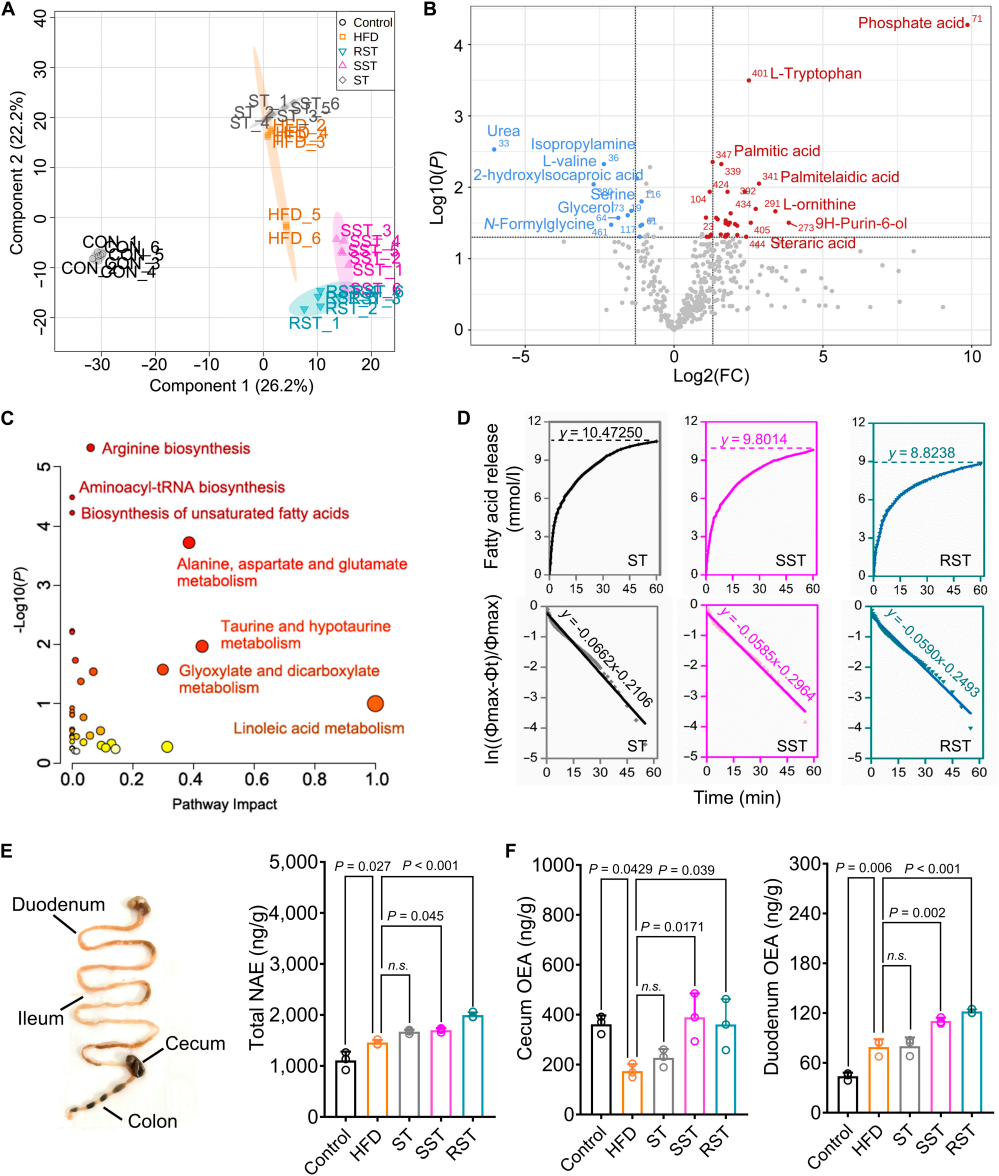
Alleviation of lipid metabolism and FAAH metabolites (NAE) in the intestine of DMS-treated mice. (A) PLS-DA plot based on the intestinal metabolite profiles of different groups (*n* = 6). (B) Volcano plot showing the Log2FC of significantly changed metabolites with DMS treatment (HFD/RST) (*n* = 6). (C) Metabolic pathway analysis organized by pathway enrichment analysis (*P* values) and pathway topology analysis (pathway impact). (D) DMS decreases fatty acid release rate and release rate constant during in vitro digestion. (E and F) Changes in the total N-acylethanolamine (NAE) level (E) and OEA levels in the cecum and duodenum (F). Significant differences among 3 or more mean values were determined by one-way ANOVA with Tukey’s multiple comparisons test.

We further employed untargeted and targeted metabolomics to investigate whether FAAH-1 metabolites are involved in the beneficial effects of DMS. The intestinal metabolites between the control, HFD, and RST/SST groups were separately distributed (Fig. 5A). Significantly differentiated metabolites were identified through biomarker analysis and illustrated using a volcano plot (Fig. 5B). As expected, many fatty acid metabolites were found to be down-regulated in the DMS group (HFD/RST), such as palmitic acid. Pathway analysis revealed significant changes in lipid metabolism pathways, including biosynthesis of unsaturated fatty acids and linoleic acid metabolism (Fig. 5C). Informed by the lower abundance of fatty acids in the intestine and the increased fecal triacylglycerol level (Fig. 3D), we hypothesize that intestinal triacylglycerol hydrolysis might be affected by DMS. In vitro pancreatic lipase digestion was then performed to verify the triacylglycerol hydrolysis behavior. As shown in Fig. 5D, the maximum fatty acid release after hydrolysis in the ST group was significantly higher than that in the DMS group. The first-order kinetic curves also indicated that DMS treatment decreased release rate constants (Fig. 5D). This result confirmed a lower degree and speed of intestinal triacylglycerol hydrolysis, indicating less efficient lipid hydrolysis in the intestine of DMS-fed mice, leading to reduced fatty acid abundance in RST and SST groups.

Given that OEA is an essential regulator of lipid metabolism through blocking lipolysis in the intestine [28–30], we sought to confirm if the reduced triacylglycerol hydrolysis was due to an increased OEA level resulting from FAAH inhibitory effects by DMS. We measured N-acylethanolamine (NAE) metabolites in different parts of the intestine (Fig. 5E). Indeed, the total NAEs were significantly increased in the DMS groups (Fig. 5E); in particular, the OEA level was lower in both the cecum and duodenum (Fig. 5F). Therefore, we again confirmed that DMS improves lipid metabolism by enhancing the level of FAAH metabolites (OEA) in the mouse model.

## Discussion

The incorporation of a hydroxyl group (3β-OH) into the phytosterol structure plays a crucial role in bioreceptor recognition. Steric hindrance caused by adjacent CH_3_ groups significantly influences the physicochemical behavior of DMS [31–33]. Specifically, these CH_3_ substituents can induce the rotation of the 3β-OH group, facilitating the formation of hydrogen bonds with amino acid residues near the targeted protein [34]. This altered polarity, resulting from the presence of CH_3_ groups, effectively distinguishes DMS from 4-demethylsterols (Fig. 3A). To our knowledge, we present the first report on the purification of DMS from dietary sources using MPLC, a methodology also applicable for scaling up DMS preparation.

*C. elegans* serves as a well-established model in biomedical research, particularly for studies related to metabolic disorders [35]. Using the *C. elegans* model, we demonstrated that DMS significantly reduced fat accumulation while enhancing locomotion (Fig. 1C to F). Given the strong correlation between fat accumulation, locomotion, and FAAH-1 inhibition activity [25], we hypothesize the involvement of the FAAH-1-mediated pathway in reducing fat content and improving motility. To validate this hypothesis, we quantified the levels of FAAH-1 hydrolysis substrate (OEA) and product (OA). OEA levels were elevated, whereas OA levels decreased (Fig. 2A). Furthermore, the effects on fat content and locomotion were abolished in the faah-1 mutant. Reduced in vivo FAAH-1 expression levels and inhibition of purified FAAH-1 enzyme activity in vitro confirmed the inhibitory activity of DMS on FAAH-1 protein.

To explore whether all DMS compounds exhibit inhibitory effects on FAAH-1, we conducted molecular docking screening for phytosterols. Docking results revealed evident binding interactions between DMS and the catalytic residue (Ser241) in the FAAH-1 protein [36], primarily driven by hydrogen bonding. These findings affirmed that FAAH-1 is the target inhibited by DMS. The proposed mechanism suggests that the presence of 2 CH_3_ groups diminishes the polarity of the 3β-OH in the A ring of DMS, whereas 4-demethylsterols exhibit exposed 3β-OH polarity (Fig. S2). Prior to accessing the catalytic center (Ser241) of FAAH-1, polar amino acid residues on the entrance surface act as a barrier, capturing 4-demethylsterols due to their higher polarity. DMS is not captured because of its less exposed 3β-OH polarity, allowing DMS to be recognized by the Ser241 residue through hydrogen bonding interactions. Consequently, the capacity of FAAH-1 for NAE hydrolysis is diminished. The purified FAAH-1 protein inhibitory assay validated the docking results (Fig. 2F). These collective findings confirm that DMS possesses FAAH-1 inhibitory activity attributable to the additional CH3 substitutes adjacent to the 3β-OH group.

Subsequently, our investigation aimed to elucidate whether the FAAH-1 inhibitory function of DMS enhances fat metabolism in both cellular and mouse models. Unlike 4-demethylsterols, DMS did not demonstrate a significant cholesterol-lowering effect (Fig. S3). This observation may be attributed to the structural dissimilarity between 4-demethylsterols and cholesterol, rendering DMS ineffective in interfering with micellar formation and affecting cholesterol absorption [37]. DMS possesses 2 additional CH_3_ groups adjacent to the 3β-OH group, whereas 4-demethylsterols differ mainly in their side chain (Fig. 3D). Notably, compared to 4-demethylsterols, DMS intervention resulted in a significant reduction in body weight (Fig. 4A to C) and enhanced triacylglycerol excretion (Fig. 4D).

Previous literature has established a connection between the ECS function and lipolysis [29,30]. Triacylglycerol undergoes hydrolysis by lipase before absorption by intestinal cells. Thus, we conducted an in vitro digestion model to explore the impact of different phytosterols on triacylglycerol hydrolysis during intestinal digestion. As expected, the rate of fatty acid release was notably slower in the DMS-treated groups compared to those treated with 4-demethylsterols (Fig. 5D). A previous study demonstrated that the release rate of fatty acids decreased with an increased proportion of DMS ferulate [38], suggesting a potential inhibitory activity of DMS on pancreatic lipase during triacylglycerol hydrolysis. Consistent with this, we observed a significant reduction in eWAT weight following DMS treatment, which notably increased triacylglycerol excretion. The elevation in triacylglycerol excretion could lead to reduced fat accumulation in eWAT. However, the detailed interaction between DMS and lipase activity remains elusive.

Furthermore, DMS may also exhibit anti-inflammatory properties, as indicated by hepatic histological analysis revealing less white vacuolation and microvesicular steatosis in the intervention groups (Fig. S3C). It has been demonstrated that DMS can mitigate hepatotoxicity by reducing oxidative stress and inflammatory markers [39]. Oxidative stress and inflammation are pivotal in up-regulating fatty acid synthesis; elevated fatty acid levels can further exacerbate oxidative stress and inflammation, establishing a feedback loop that influences fat accumulation [40]. Overall, the intricate interplay between DMS and the inflammatory response warrants further investigation.

To investigate the impact on intestinal lipid absorption, we employed untargeted and targeted metabolomics to assess changes in intestinal metabolites induced by DMS. We observed a significant reduction in intestinal fatty acid levels following DMS treatment (Fig. 5B). OEA, a key regulator of energy homeostasis, inhibits lipogenesis processes closely associated with lipid lipolysis and desaturation [28–30]. Total NAE concentrations and ratios are indicative biomarkers for obesity and lipid dysmetabolism [41]. We hypothesized that DMS might affect NAE production due to its inhibitory effects on FAAH-1 activity. Indeed, we observed higher OEA concentrations in the duodenum and cecum of DMS-treated groups (Fig. 5). OEA plays a critical role in enhancing lipid metabolism and affecting fat accumulation [42]. For instance, the observed reduction in body weight gain in mice correlated with significant changes in OEA levels specifically in the small intestine, but not in the brain or muscle [43]. Thus, the observed reduction in fat accumulation attributed to DMS may be linked to elevated OEA levels resulting from FAAH-1 inhibition.

**Fig. 6.**
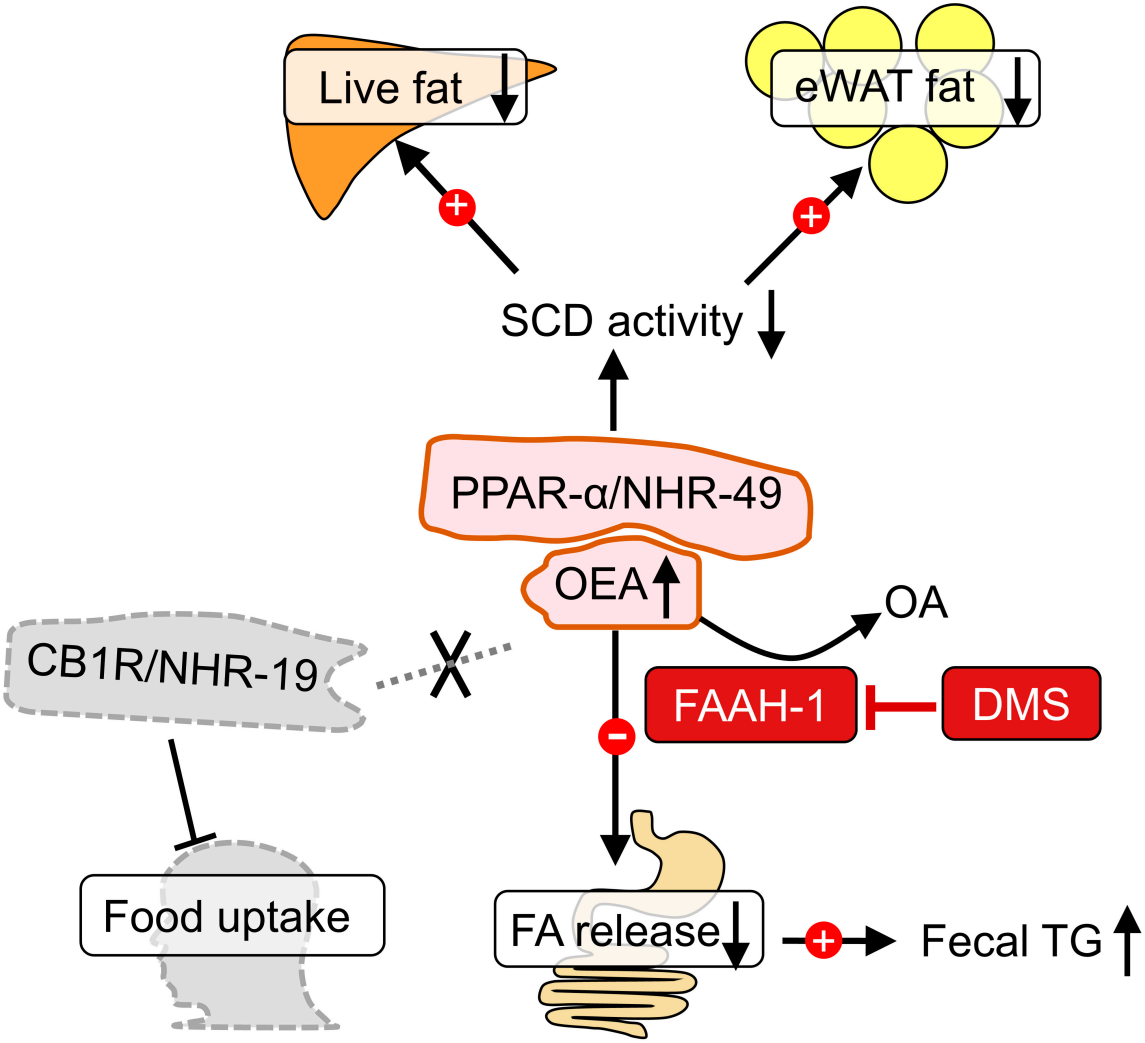
Proposed mechanism of DMS-mediated fat reduction through FAAH-1 inhibition. FAAH-1 plays a critical role in hydrolyzing OEA into OA. DMS effectively inhibits FAAH-1, leading to increased levels of OEA. OEA acts as an agonist for peroxisome proliferator-activated receptor α (PPAR-α) and its homolog, nuclear hormone receptor-49 (NHR-49) in *C. elegans*. The elevated OEA concentration activates PPAR-α, promoting the β-oxidation pathway and reducing fat levels by down-regulating stearoyl-CoA desaturase (SCD) expression in the liver and adipose tissues. Furthermore, increased OEA inhibits pancreatic lipase activity, reducing intestinal fatty acid release and promoting fecal TG excretion. The FAAH-1-mediated lipid-lowering mechanism is independent of CB1R or food uptake.

The mechanism underlying the lipid-lowering activity of DMS through FAAH-1 inhibition is illustrated in Fig. 6. By inhibiting FAAH-1 activity, DMS increases the levels of OEA, which acts as an agonist for peroxisome proliferator-activated receptor α (PPAR-α) [44]. In *C. elegans*, nuclear hormone receptor-49 (NHR-49) serves as a homolog of PPAR-α. Our research has shown that reduced NHR-49 plays a crucial role in the fat-lowering activity of DMS [8], suggesting that the interaction between OEA and NHR-49 leads to decreased stearoyl-CoA desaturase (SCD) activity. Activation of PPAR-α affects the expression of stearoyl-CoA desaturase (SCD) and reduces fat synthesis in adipose tissue in mice [45]. Moreover, the increased OEA level also activates PPAR-α, enhancing liver mitochondrial respiration for β-oxidation [46,47], which consequently lowers triglyceride (TG) levels. Additionally, previous studies indicate that increased OEA levels can suppress lipase activity [48], aligning with our observations of inhibited TG hydrolysis in the small intestine and increased fecal TG excretion. Importantly, these effects appear to be independent of food intake.

In summary, our study highlights the superior anti-obesity effects of dietary DMS compared to 4-desmethylsterols through the inhibition of FAAH-1 activity. We establish a crucial link between DMS and the ECS in reducing fat accumulation, presenting DMS as a promising dietary intervention strategy for combating diet-induced obesity. DMS derived from natural foods or through nutrient supplementation holds significant potential for use in functional nutraceutical development, given its widespread presence in vegetable oils and nuts. Future research should focus on delineating specific differences among various DMS compounds and expanding their applications in the food industry. Clinical investigations are essential to fully elucidate the therapeutic potential and optimal dosing strategies of DMS across diverse health conditions. These efforts will contribute to advancing our understanding of DMS as a promising candidate for addressing obesity and related metabolic disorders.

## Materials and Methods

### Chemical reagents

Different sterols were purchased from Sigma-Aldrich (Shanghai, China) and ChemFaces Co., Ltd. (Wuhan, China). The FAAH inhibitor screening assay kit (Item No. 10005196) was obtained from Cayman Chemical (Ann Arbor, MI). The FAAH ELISA kit was purchased from Jianglai Biological Technology Co., Ltd. (Shanghai, China). Fatty acid monoethanolamides (>95%) were synthesized according to our previously established method [49]. Other reagents and organic solvents were of analytical grade and were purchased from Sigma-Aldrich or Fisher Scientific Inc.

### *C. elegans* model

Details about the *C. elegans* strains can be found in Table S1. The worms were cultured on nematode growth media (NGM) agar plates at 20 °C. Synchronization was conducted by treatment with a lysis solution composed of 1.25 ml of NaOH (10 mol/l), 5 ml of NaClO (5%), and 17.5 ml of water. Phytosterol feeding followed established protocols from previous studies [8,25]. Prior to plating L1 worms, different sterols were dissolved in ethanol with the assistance of ultrasound, resulting in a stock concentration of 10 mg/ml. These solutions were subsequently diluted with sterile water to achieve a final working concentration of 100 μg/ml. Each treatment consisted of a mixture of 100 μl of phytosterols and 100 μl of concentrated OP50, which was then spread onto a 10-ml NGM plate. The final treatment concentration was 50 μg/ml. For locomotion and functional evaluations of DMS, a basal cholesterol concentration of 5 μg/ml was maintained in NGM. After 12 h, the growth of OP50 was arrested using ultraviolet radiation. Upon reaching adulthood, the worms were collected for analysis. Each measurement was independently repeated 3 times.

### Sterol-depleted experiments

Sterol-depleted conditions were performed following a previous study with slight modifications [23]. Third-generation worms, depleted of cholesterol, were transferred to 60-mm-diameter plates containing different sterols at a concentration of 5 μg/ml. In this setup, agarose was used as a replacement for agar powder. Peptone was pretreated with a chloroform and methanol mixture (1:2, v/v) to remove sterols. After 2 generations of sterol depletion, a single worm from the third generation was selected and incubated at 20 °C. The worm was subsequently analyzed and imaged after a 3-day incubation period. Cholesterol and ethanol were used as positive control and vehicle, respectively. Cholesterol was not added to the basic culture when *C. elegans* was fed with other phytosterols. The final concentration of DMS was the same as cholesterol (5 μg/ml). The analysis of phytosterols was performed as previously described using gas chromatography–mass spectrometry (GC-MS) [50].

### Oil red O staining

Oil Red O staining was performed following a previously established protocol [51]. Briefly, worms were fixed in 1% paraformaldehyde prepared in PBS for 30 min and subjected to 3 cycles of freezing and thawing. After washing with PBS, they were dehydrated in 60% isopropanol for 15 min. The worms were then stained with 0.5 ml of 60% Oil Red O solution for 4 h. Subsequently, the samples were washed with PBS 3 times and mounted for imaging using a Nikon Eclipse Ti-S microscope.

### In vitro FAAH activity assay

Screening of phytosterols for their potential to inhibit FAAH activity was conducted following the instructions provided by Cayman Chemical (Ann Arbor, Michigan, USA) and a reported method [52]. The results were compared with those obtained using the standard inhibitor (JZL195).

### Mouse model

Forty male mice (C57BL/6J) were obtained from Vital River Laboratory Animal Technology Co., Ltd., in Beijing, China. The experiment was conducted in accordance with the ethical guidelines of Jiangnan University (Approval No. JN.No20191230c0500615[400]). All mice were provided with ad libitum access to food and water and were housed in an environmentally controlled facility with a temperature range of 22 °C to 24 °C, humidity maintained at 70% to 75%, and a 12-h light–dark cycle. After 1 week of acclimation, the mice were divided into 5 groups, with 8 mice in each group housed in 2 separate cages. These groups were fed different diets, including a low-fat diet (Control), an HFD, an HFD enriched with 4-demethylsterols (ST, 1%), an HFD enriched with rice bran 4,4-dimethylsterols (RST, 1%), and an HFD enriched with shea nut 4,4-dimethylsterols (SST, 1%). The HFD was modified from a Western-style diet with 60% of calories from fat (D12492), while the control diet was modified from AIN93A, with fat contributing to 10% of the total calories. All diets were prepared by Jiangsu Xietong Pharmaceutical Bioengineering Co., Ltd., in Jiangsu, China. The composition of the diets can be found in Table S2. Body weight and food intake were measured weekly. Fecal samples from each animal were collected every week. After a 12-week intervention, the mice were sacrificed, and blood samples were centrifuged to collect serum. Tissues for biochemical analysis were collected, weighed, immediately frozen in liquid nitrogen, and stored at −80°C.

### Biochemical analysis

Serum lipid parameters were determined using a fully automatic biochemistry analyzer (Model 7600, Hitachi, Japan). Tissue lipid parameters, including cholesterol and triacylglycerol, were analyzed through enzymatic hydrolysis (Jiancheng Bioengineering Institute, Nanjing, China). Absorbance readings were obtained using a SpectraMax iD5 microplate reader (Molecular Devices, Sunnyvale, CA). All calculations were normalized by protein levels. The identification of fatty acid methyl esters (FAME) was analyzed by GC-MS [53].

### Untargeted GC-MS analysis

GC-MS-based untargeted metabolomics analysis was conducted following a previously published protocol [54]. Approximately 50 mg of each freeze-dried sample was placed into a 2-ml tube with 400 μl of methanol-chloroform (3:1, v/v). Then, 20 μl of internal standards containing 2-chloro-d-phenylalanine (20 μg/ml), heptadecanoic acid (100 μg/ml), and 5α-cholestan-3β-ol (100 μg/ml) was added. After homogenization for 5 min at 65 Hz (Scientz-48, Xinzhi Biotech, Ningbo), the upper layer was obtained by centrifugation at 12,000 rpm for 15 min at 4 °C. A 10-μl volume was taken from each sample to create quality control samples. After drying under nitrogen, the samples were dissolved in 80 μl of methoxyamine hydrochloride solution in pyridine (20 mg/ml) and incubated for 20 min at 80 °C. The derivatization process was carried out with BSTFA and 1% TMCS (100 μl) at 70 °C for 1 h. Metabolites were analyzed by GC-MS using a DB-5 capillary column (30 m × 0.25 mm × 0.25 μm, Agilent). Peak identification was performed using a database from the National Institute of Standards and Technology (NIST) library. The data were processed using MetaboAnalyst 5.0.

### Targeted UPLC-Q-TOF/MS analysis

Targeted NAE lipids were quantified using ultra-performance liquid chromatography (UPLC) coupled to quadrupole time-of-flight (Q-TOF) mass spectrometry (MS) [55]. The extracted lipid was re-suspended in 100 μl of acetonitrile. A BEH C18 column (50 mm × 2.1 mm × 1.9 μm, Waters) was employed with the following gradient program: 0 min (water/acetonitrile 4:6); 0.5 min (water/acetonitrile 4:6); 6.5 min (water/acetonitrile 2.5:7.5); 7.5 min (water/acetonitrile 2.5:7.5); 8 min (water/acetonitrile 4:6). The column temperature was maintained at 45 °C, and the autosampler tray temperature was set to 45 °C. Electrospray ionization (ESI) in positive-ion mode was used. The capillary voltage was set to 3.0 kV, and the desolvation temperature was maintained at 400 °C. Collision gas and desolvation gas were argon and nitrogen, respectively. Detection of NAE lipids was performed using multiple reaction monitoring (MRM) with the following transitions: *m*/*z* 300→62 for PEA; *m*/*z* 324→62 for LEA; *m*/*z* 326→62 for OEA; *m*/*z* 326→62 for SEA; *m*/*z* 346→62 for EPEA; and *m*/*z* 348→62 for AEA. The concentration ratios of the NAEs were determined by calculating the peak-area ratios of the analytes to the external standards.

### Statistical analysis

We conducted unpaired 2-tailed *t* tests for 2-group comparisons and used ANOVA followed by unpaired 2-tailed *t* tests or Tukey's tests for multiple groups. All data are presented as mean ± SEM, and sample sizes and statistical details are described in the figure legends.

## Data Availability

Other experimental details are presented in the Supplementary Materials. Additional data related to this paper can be requested from the authors.

## Supplementary Materials

Supplementary 1 Figs. S1 to S3 Tables S1 and S2 Supplemental Experimental Details
