# Supplementary material for: 4,4-Dimethylsterols Reduces Fat Accumulation via Inhibiting Fatty Acid Amide Hydrolase In Vitro and In Vivo
Source: Research (Wash D C). 2024 May 29;7:0377. doi: 10.34133/research.0377 (PMC11134202; doi:10.34133/research.0377)
Supplement: Supplementary 1 — Figs. S1 to S3 Tables S1 and S2 Supplemental Experimental Details [file research.0377.f1.zip › Revised_Supplementary Information.docx]

**Supplementary Information**

**4,4-Dimethylsterols reduces fat accumulation via inhibiting fatty acid amide hydrolase in vitro and in vivo**

Tao Zhang, Liangliang Xie, Yiwen Guo, Yandan Wang, Zhangtie Wang, Xin Guo,

Ruijie Liu, Ming Chang, Qingzhe Jin, Xingguo Wang

Correspondence to: [chang@jiangnan.edu.cn](mailto:chang@jiangnan.edu.cn); [wangxg1002@gmail.com](mailto:wangxg1002@gmail.com)

This PDF includes:

- Supplementary Figures 1-3

- Supplementary Table 1-2

- Supplemental experimental details


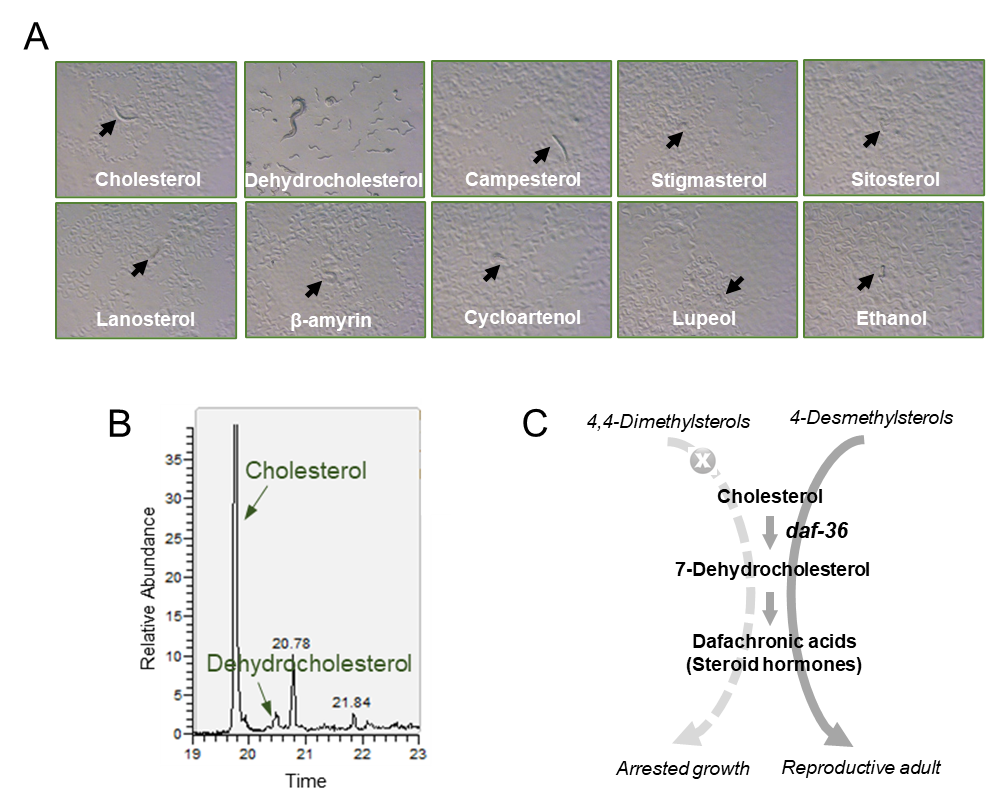


**Figure S1.** **7-Dehydrocholesterol supports population growth in cholesterol-depleted *daf-36* mutant worms.** (A) Among the tested DMS (lanosterol, β-amyrin, cycloartenol, lupeol), only 7-dehydrocholesterol supports population growth in the third generation of cholesterol-depleted *daf-36* mutant worms. (B) GC-MS analysis confirms cholesterol synthesis in *daf-36* mutant worms fed with 7-dehydrocholesterol. (C) The metabolic pathway of 4-desmethylsterols leading to cholesterol synthesis occurs upstream of the conversion from cholesterol to 7-dehydrocholesterol. This pathway highlights the essential role of 4-desmethylsterols as intermediates in cholesterol biosynthesis. Notably, DMS cannot be converted into cholesterol.


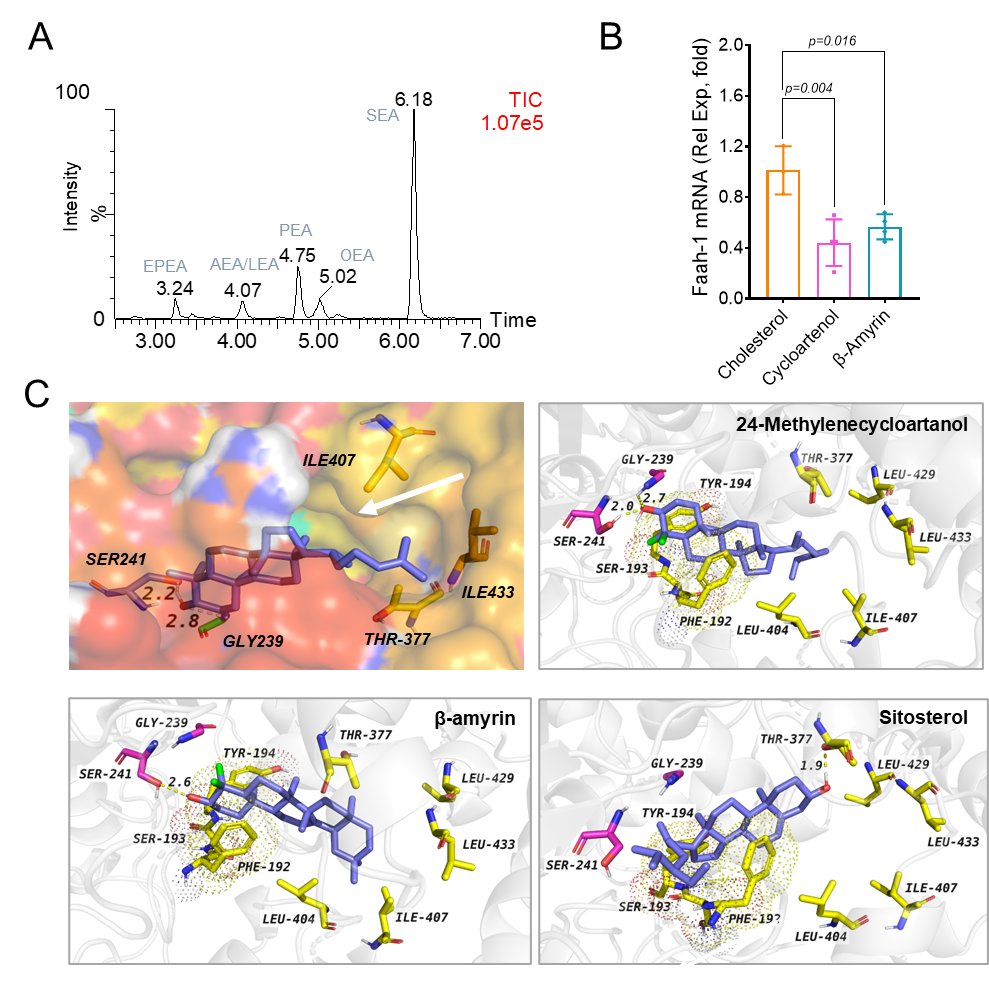


**Figure S2. Effects of DMS on NAE levels, *faah-1* mRNA transcription in *C. elegans*, as well as its interaction with FAAH-1 protein *in vitro*.** (A) LC-MS analysis of the profile of N-acylethanolamines (NAEs) in the total ion chromatograph. (B) Relative expression levels of *faah-1* mRNA in wild-type *C. elegans* after DMS treatment. (C) Molecular docking analysis of the interaction between FAAH-1 protein and cycloartenol and stigmasterol (4-demethylsterols), with the entrance surface indicated by the white arrow.


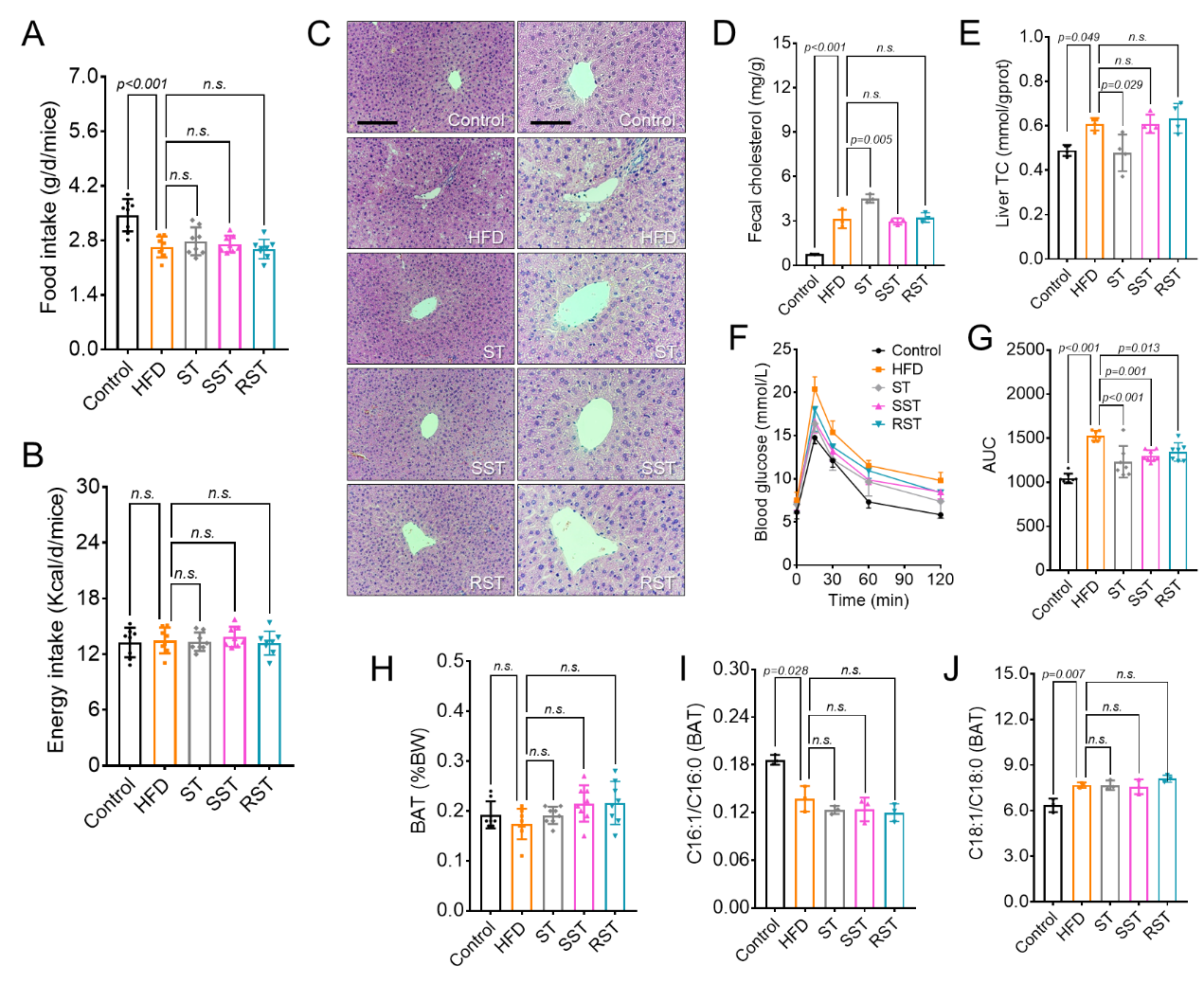


**Figure S3.** **DMS decreases fat accumulation through enhancing triacylglycerol excretion and inhibiting lipase activity in mice.** (A) Food intake and (B) energy intake of mice from different treatments. (C) Representative hepatic tissue H&E staining (scale bars: 200 µm and 100 µm, respectively). (D) Fecal TG excretion, (E) Liver TC, (F-G) Blood glucose levels and area under the curve. (H-J) Changes in brown adipose tissue (BAT) weight (H), C18:1/C18:0 ratio (I), and C16:1/C16:0 ratio (J) in BAT. Significance among three or more mean values was determined using one-way ANOVA with Tukey’s multiple comparisons test.

**Table S1.** ***C. elegans* strains used in this study.**

| Strain | Genotype | Source | Ref. |
| --- | --- | --- | --- |
| N2 | Wild type | Caenorhabditis Genetics Center (CGC) |  |
| AA292 | daf-36(k114) V. | Caenorhabditis Genetics Center (CGC) | [1] |
| TM5011 | faah-1 mutants IV. | Prof. Hisamoto’s Lab | [2] |
| Pfaah::GFP | Pfaah-1::GFP | Prof. Hisamoto’s Lab | [3] |
| ZXW618 | Pdhs-3::dhs-3::GFP | Prof. Liu’s Lab | [4] |

**Table S2. Compositions of the diet of HFD mice model.**

|  | Control | HFD | ST | RST | SST |
| --- | --- | --- | --- | --- | --- |
| Total fat (g/100 g) | 5.2 | 37.1 | 36.9 | 37.3 | 36.5 |
| Lauric acid (C12:0) | 0.73 | 0.23 | 0.27 | 0.25 | 0.29 |
| Myristic acid (C14:0) | 0.60 | 2.28 | 2.25 | 2.35 | 2.58 |
| Palmitic acid (C16:0) | 23.74 | 32.65 | 32.78 | 32.07 | 32.28 |
| Stearic acid (C18:0) | 3.47 | 8.35 | 8.65 | 8.78 | 8.14 |
| Oleic acid (C18:1) | 23.47 | 30.05 | 28.16 | 28.44 | 28.32 |
| Linoleic acid (C18:2) | 38.43 | 17.58 | 17.56 | 17.75 | 17.11 |
| Linolenic acid (C18:3) | 3.62 | 1.22 | 0.31 | 0.34 | 0.30 |
| Total cholesterol (mg/100 g) | 0.54 | 79.30 | 79.16 | 78.84 | 77.26 |
| Total phytosterols (mg/g) | 4.61 | 0.77 | 10.20 | 9.70 | 10.05 |

**Supplemental Experimental Details**

**Microscopy imaging**

Worms were immobilized on 2% agarose pads containing 0.5% NaN3 as an anesthetic. Adult worms were observed and imaged on the third day after L1 arrest using a fluorescence microscope (Nikon Eclipse Ti-S, Japan).

**Tracking behavior analysis**

The behavior of adult worms on the third day was recorded at 30-second intervals using an Olympus SZX16 optical microscope equipped with an MSI digital camera. Movement speed was determined using WormLab software (MBF Bioscience, Williston, USA) following the provided instructions.

**In vivo ELISA assay**

After treatment with various phytosterols for 3 days, worms were collected for FAAH activity analysis. FAAH activity was determined using the *C. elegans* enzyme-linked immunosorbent assay (ELISA) kit from Jianglai Biological Co., Ltd. (Shanghai, China), following the provided protocols.

**Quantitative RT-qPCR**

Total RNA was extracted using RNAiso Plus reagent (Takara, China). The isolated RNA was reverse-transcribed into cDNA using an RT Master Mix (Vazyme, Nanjing, China). Quantitative PCR (qPCR) was performed using SYBR Green Master Mix (Vazyme, Nanjing, China) according to the manufacturer’s instructions. The relative mRNA level was normalized to the internal control gene (act-1). The following primer sequences were used: faah-1_fw: GGAGCCGGAGGTTCATTGATTG, faah-1_rev: ACCACCACCACGATGAGCAAATC. act-1_fw: CCCACTCAATCCAAAGGCTA, act-1_rev: ATCTCCAGAGTCGAGGACGA.

**Molecular docking study**

The X-ray crystal structure of FAAH-1 (PDB ID: 2VYA) was downloaded from the Protein Data Bank[5]. The structures of various phytosterols were generated using Chem3D Ultra 8.0. To prepare for docking simulations, 2VYA was processed by removing ligands, water molecules, and co-crystallized metals. Subsequently, polar hydrogen atoms and partial charges were added to the macromolecule. Docking calculations were performed using AutoGrid4 and AutoDock4, and MGL Tools version 1.5.6. Ser241 was selected as the flexible residue. Flexible-ligand docking was specifically performed for phytosterols with a grid box size of 60 × 60 × 60 Å, centered at the center of 2VYA (x=47.753; y=2.764; z=-2.822).

**Purification and identification of phytosterols**

The unsaponifiable matter was extracted by saponification using 1 M KOH in ethanol. Liquid-liquid extraction followed the method described by the Association of Official Agricultural Chemists (AOAC)[6]. Purification of different phytosterols was accomplished using preparative Medium Pressure Liquid Chromatography (MPLC) with a silica column (40 μm, 40 g, Grace) in flash mode. The unsaponifiable matter in a hexane solution was injected and eluted according to the following program: Initially, 12 mL of the sample was eluted using ethyl acetate as solvent A (ranging from 20% to 70%) and hexane as solvent B (ranging from 80% to 30%) from 0 to 15 minutes, followed by a 10-minute maintenance period. The flow rate was set at 15 mL/min, and fractions were automatically collected by monitoring the targeted peaks. After drying under reduced pressure, the collected fractions (in 100 μL of dichloromethane) were analyzed for purity verification using thin-layer chromatography (Gel 60, 20 cm × 10 cm, 0.25 mm; Merck, Darmstadt). The composition of each corresponding fraction was determined using HPLC-ELSD.

**Histology**

In brief, fresh epididymal fat and liver tissues were immersed in 4% paraformaldehyde. Visceral sections were then observed after hematoxylin-eosin (H&E) staining using an inverted microscope (Nikon Eclipse Ti-S, Japan), following established procedures[7].

**Fatty acid analysis**

Total lipids were extracted following a previously reported method[8]. Freeze-dried samples were homogenized with chloroform/methanol (1.2 mL, 2:1, v/v), followed by the addition of water (0.6 mL). After centrifugation for 5 minutes at 8000 rpm, the lower organic lipid phase was collected. The remaining upper phase was subjected to re-extraction. The organic phases were combined, dried under nitrogen, and dissolved in n-hexane, with the addition of 50 μg of heptadecanoic acid as an internal standard. Each lipid class was methylated using 0.5 M NaOH in methanol at 60°C for 30 minutes. The resulting fatty acid methyl esters were extracted using hexane and subsequently dried under nitrogen. Fatty acid methyl esters were analyzed by gas chromatography (GC)..

**In vitro intestinal digestion**

Simulated intestinal digestion was conducted following a previously published protocol. In brief, 30 mL of oil (glycerol trioleate) mixed with different phytosterols (1%) was emulsified. The crude emulsion was adjusted to pH 7.0 and loaded into a 100 mL glass reaction vessel with a water bath set at 37°C. Next, 1.5 mL of a pre-mixed buffer solution containing CaCl2 and NaCl was added with continuous stirring, followed by 3.5 mL of bile salt solution (5%, w/v). After adjusting the pH to 7.0, porcine pancreatic lipase (24 mg/mL, 2.5 mL) in a buffer solution was added. An automatic titration device recorded the consumption of NaOH solution (0.15 mol/L) during the digestion process.

$$M_{FFA}=\frac{V_{NaOH}\times m_{NaOH}}{V_{e}}$$

where,

*M_FFA_* represents the amount of released fatty acids (μmol/mL),

*V_NaOH_* denotes the consumed volume of NaOH (mL),

*m_NaOH_* represents the molar mass of NaOH,

*V_e_* signifies the digestion volume of the phytosterol-enriched oil emulsion.

*ln[(Φ_max_－Φ_t_)/Φ_max_]=-kt+b*

where,

*Φ_max_* is the total fatty acid release content,

*k* is the first-order rate constant (s^-1^),

*t* refers to the digestion time (s).

The total fatty acids release content (*Φ_max_*) was determined by integrating the fatty acid release curve.

**References:**

1. Motola DL, Cummins CL, Rottiers V, Sharma KK, Li T, Li Y, et al. Identification of Ligands for DAF-12 that Govern Dauer Formation and Reproduction in *C. elegans*. Cell. 2006;124(6):1209-23. doi: <http://doi.org/10.1016/j.cell.2006.01.037>.

2. Pastuhov SI, Fujiki K, Nix P, Kanao S, Bastiani M, Matsumoto K, et al. Endocannabinoid-Goα signalling inhibits axon regeneration in *Caenorhabditis elegans* by antagonizing Gqα-PKC-JNK signalling. Nature Communications. 2012;3(1):1136. doi: <https://doi.org/10.1038/ncomms2136>.

3. Harrison N, Lone MA, Kaul TK, Reis Rodrigues P, Ogungbe IV, Gill MS. Characterization of N-Acyl Phosphatidylethanolamine-Specific Phospholipase-D Isoforms in the Nematode *Caenorhabditis elegans*. PLOS ONE. 2014;9(11):e113007. doi: <https://doi.org/10.1371/journal.pone.0113007>.

4. Zhang P, Na H, Liu Z, Zhang S, Xue P, Chen Y, et al. Proteomic Study and Marker Protein Identification of *Caenorhabditis elegans* Lipid Droplets. Molecular & Cellular Proteomics. 2012;11(8):317-28. doi: <http://doi.org/10.1074/mcp.M111.016345>.

5. Mileni M, Johnson DS, Wang Z, Everdeen DS, Liimatta M, Pabst B, et al. Structure-guided inhibitor design for human FAAH by interspecies active site conversion. Proc Natl Acad Sci USA. 2008;105(35):12820. doi: <https://doi.org/10.1073/pnas.0806121105>.

6. Firestone D. Official Methods and Recommended Practices of the AOCS Analytical methods. 5th ed: American Oil Chemists' Society; 2009.

7. Wang C, Niederstrasser H, Douglas PM, Lin R, Jaramillo J, Li Y, et al. Small-molecule TFEB pathway agonists that ameliorate metabolic syndrome in mice and extend *C. elegans* lifespan. Nat Commun. 2017;8(1):2270. doi: <https://doi.org/10.1038/s41467-017-02332-3>.

8. Folch J, Lees M, Sloane Stanley GH. A simple method for the isolation and purification of total lipides from animal tissues. J Biol Chem. 1957;226(1):497-509. Epub 1957/05/01. doi: <https://dx.doi.org/10.17504/protocols.io.bbu9inz6>.
