# Supplementary figures and images for: 4,4-Dimethylsterols Reduces Fat Accumulation via Inhibiting Fatty Acid Amide Hydrolase In Vitro and In Vivo
Source: Research (Wash D C). 2024 May 29;7:0377. doi: 10.34133/research.0377 (PMC11134202; doi:10.34133/research.0377)

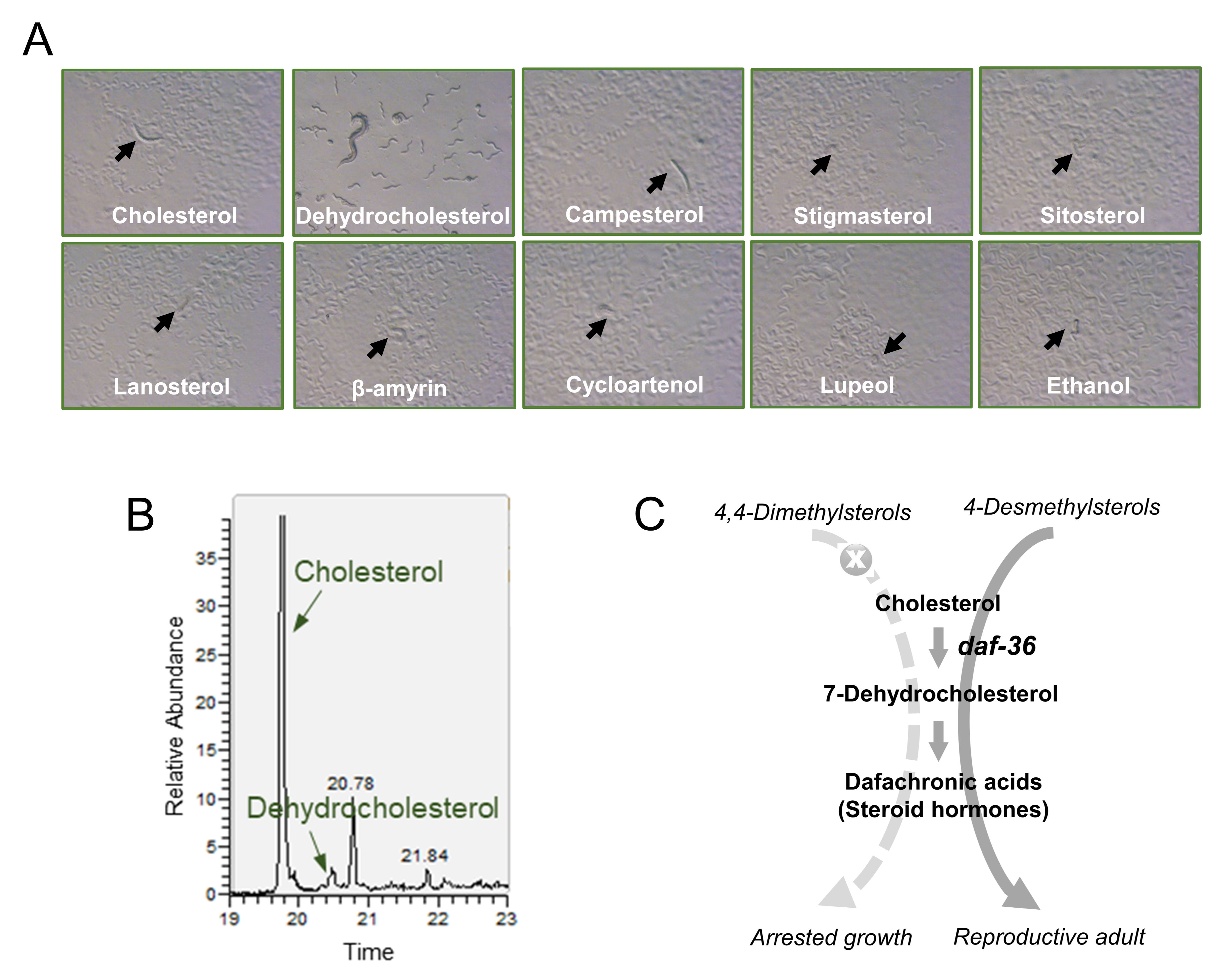

Supplement: Supplementary 1 — Figs. S1 to S3 Tables S1 and S2 Supplemental Experimental Details [file research.0377.f1.zip › Figure S1.png]

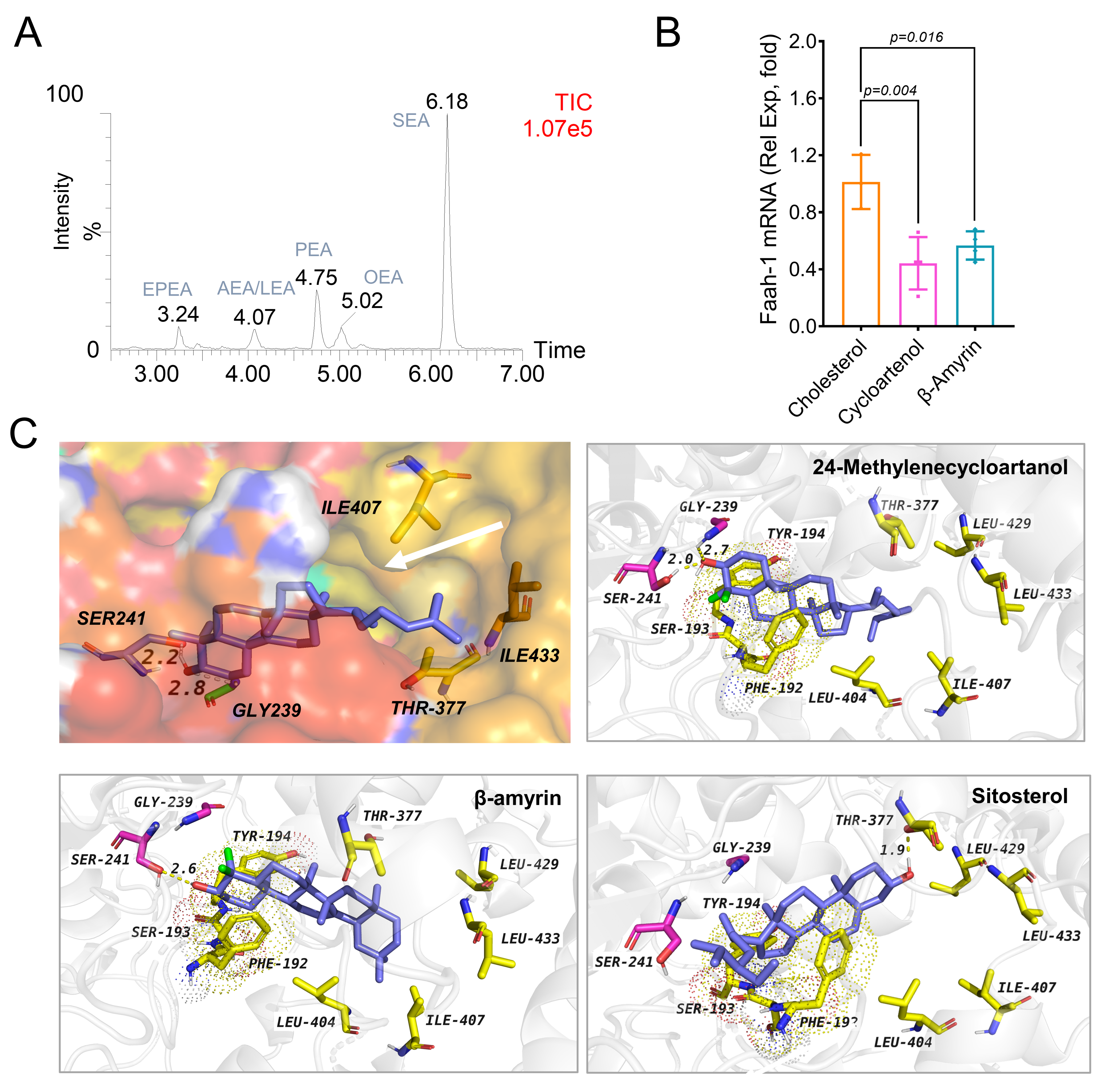

Supplement: Supplementary 1 — Figs. S1 to S3 Tables S1 and S2 Supplemental Experimental Details [file research.0377.f1.zip › Figure S2.png]

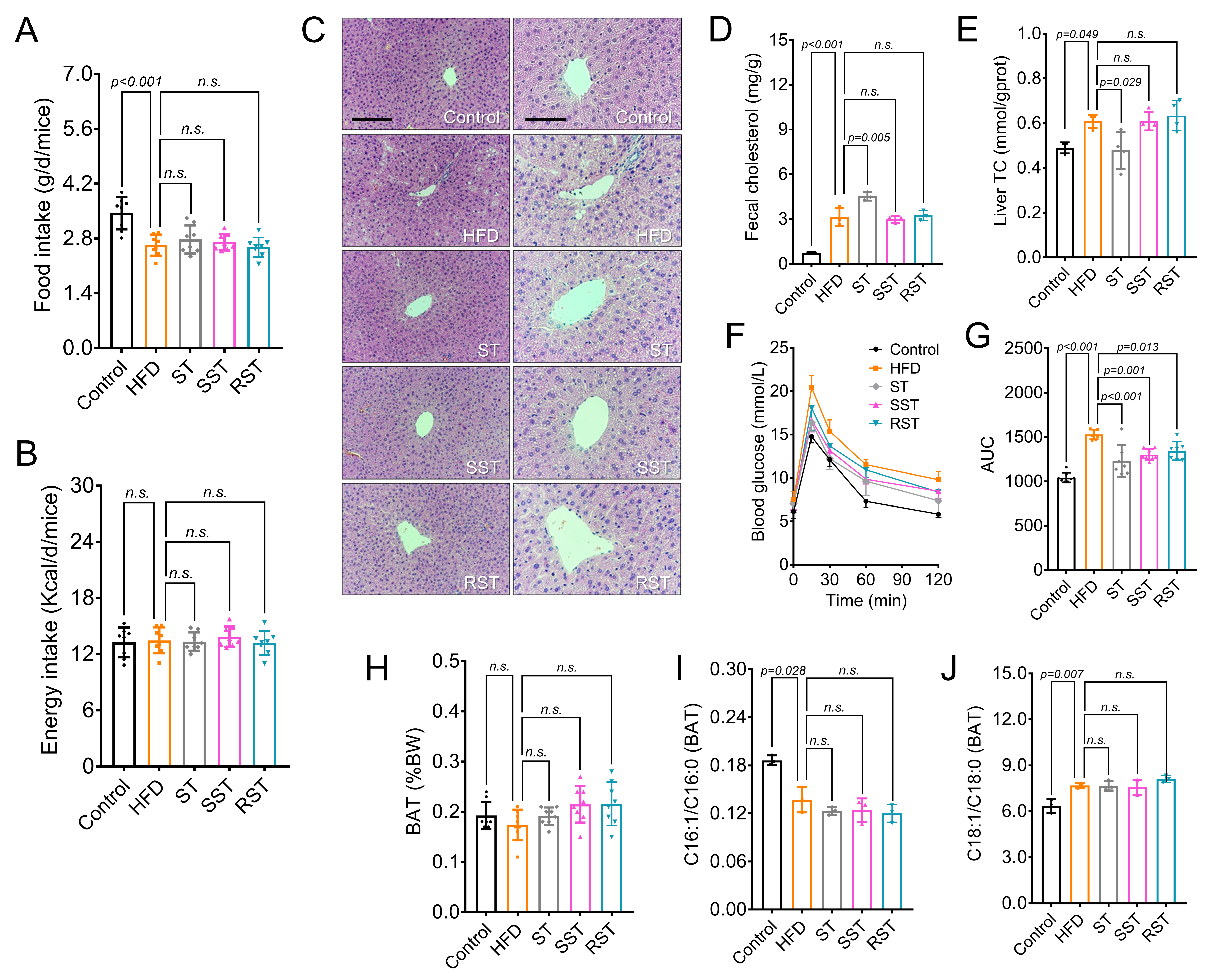

Supplement: Supplementary 1 — Figs. S1 to S3 Tables S1 and S2 Supplemental Experimental Details [file research.0377.f1.zip › Figure S3.png]
